# Specific Medication

**Published:** 1892-02

**Authors:** 


Specific Medication and Specific Medicines. Fourth Revision. With an Appendix containing the Articles published on the Subject since the First Edition. And a Report of Cases Illustrating Specific Medication. By John M Scudder, M. D., Professor of the Principles and Practice of Medicine in the Eclectic Medical Institute, etc., etc. Thirteenth Edition. Cincinnati: Published by John M. Scudder, 1890. Price, $2.50.

These two well-bound and well printed little volumes of Dr. Scudder go together. Where one is read the other should be consulted. They go hand in hand. They represent the so-called Eclectic Practice as it is taught in the parent school of Eclecticism, at Cincinnati.

As a matter of course, we, as homœopathists, can have no use for such works. With us Hahnemann's *Organon*, his *Chronic Diseases*, and *Materia Medica Pura* constitute all we need to successfully combat all physical and spiritual ailments of humanity.

We can only regret that works of this kind are in print at all, as many allopathists, by reading them, are prevented from investigating Homœopathy, thinking, as many really do, that Dr. Scudder is a homœopath. Homœopathy and Eclecticism have nothing in common. W. S.

